# Study on the Potential for Stimulating Mulberry Growth and Drought Tolerance of Plant Growth-Promoting Fungi

**DOI:** 10.3390/ijms24044090

**Published:** 2023-02-17

**Authors:** Ting Ou, Meng Zhang, Haiying Gao, Fei Wang, Weifang Xu, Xiaojiao Liu, Li Wang, Ruolin Wang, Jie Xie

**Affiliations:** State Key Laboratory of Silkworm Genome Biology, Key Laboratory of Sericultural Biology and Genetic Breeding in Ministry of Agriculture, College of Sericulture, Textile and Biomass Science, Southwest University, Chongqing 400715, China

**Keywords:** plant growth, plant growth-promoting fungi, mulberry, drought stress

## Abstract

Drought stress often leads to heavy losses in mulberry planting, especially for fruits and leaves. Application of plant growth-promoting fungi (PGPF) endows various plant beneficial traits to overcome adverse environmental conditions, but little is known about the effects on mulberry under drought stress. In the present study, we isolated 64 fungi from well-growing mulberry trees surviving periodical drought stress, and *Talaromyces* sp. GS1, *Pseudeurotium* sp. GRs12, *Penicillium* sp. GR19, and *Trichoderma* sp. GR21 were screened out due to their strong potential in plant growth promotion. Co-cultivation assay revealed that PGPF stimulated mulberry growth, exhibiting increased biomass and length of stems and roots. Exogenous application of PGPF could alter fungal community structures in the rhizosphere soils, wherein *Talaromyces* was obviously enhanced after inoculation of *Talaromyces* sp. GS1, and *Peziza* was increased in the other treatments. Moreover, PGPF could promote iron and phosphorus absorption of mulberry as well. Additionally, the mixed suspensions of PGPF induced the production of catalase, soluble sugar, and chlorophyll, which in turn enhanced the drought tolerance of mulberry and accelerated their growth recovery after drought. Collectively, these findings might provide new insights into improving mulberry drought tolerance and further boosting mulberry fruit yields by exploiting interactions between hosts and PGPF.

## 1. Introduction

Abiotic stress is a growing challenge for agriculture, which may adversely result in a decline in crop growth and productivity. Among all abiotic stresses, drought is the chief hindering factor limiting water availability, declining water potential, and closing the stomata of plants, which finally leads to lower survival rates and productivity of plants [1]. Deficiency of water within plant tissues disrupts the cellular ion balance and leads to the production of reactive oxygen species, which may further destroy cellular structures, all of which may finally impede plant development [2]. To adapt to drought stress, plants develop various strategies to balance their growth and stress responses [3]. For instance, plants usually enhance their primary root growth but inhibit lateral root growth, which helps plants acquire water from deep soils [4]. Moreover, plants also evolve a series of stress signaling pathways and produce some organic osmolytes (e.g., proline, glycine betaine, and polyamines) and antioxidant enzymes (e.g., peroxidase, catalase, superoxide dismutase, and glutathione peroxidase) in drought stress conditions, all of which can protect plant cells from osmotic stresses and scavenge reactive oxygen species accumulation [5].

Mulberry (*Morus* L.), belonging to the Moraceae family, has a long history in Asia for economical uses in silkworm cultivation and Chinese medicine supply [6]. Nowadays, fruits of mulberry emerge with significant development potential considering their nutritional and medicinal values. Mulberry fruits have a great diversity of nutritive compounds such as fatty acids, amino acids, vitamins, minerals, and bioactive compounds [7]. Importantly, partial secondary metabolites of mulberry fruit play crucial roles in anti-inflammatory, anti-atherosclerosis, immunomodulation, anti-tumor, and anti-hyperglycemic [7,8]. Large-scale mulberry plantations are traditionally located in humid areas in China since the rapid growth of mulberry trees depends on the high consumption of water [9]. However, in order to meet the increasing economic and ecological demands, many new mulberry plantations have been established in regions with poor soil, low nutrition availability, and water deficiency conditions [10,11], such as loess plateau, desertification land, and the hydro-fluctuation belt of the Three Gorges Reservoir [12,13]. The unfavorable environmental conditions, especially drought stress, are major limiting factors for the mulberry industry, which result in an underdeveloped root system, declined survival rate, and less fruit production. Generally, breeding approaches or transgenic technologies could assist plant development and improve their drought tolerance [14], but enhancement of mulberry drought resistance by these strategies may be restricted due to limited information on genetic variability and time consumption.

The interactions between plants and their beneficial fungi, especially endosphere and rhizosphere microbes, are regarded as promising strategies to stimulate plant development in adverse environments such as saline lands [15] and heavy metal-contaminated lands [16]. Many plant-associated microorganisms can benefit plant health by positively influencing plant physiology, development, and environmental adaptation [17,18]. The mechanisms employed by plant-beneficial microorganisms to promote growth are involved in various ways. Specifically, they benefit plants directly by phytohormone production and modulation such as indole-3-acetic acid (IAA) and gibberellin (GA) [19], enhancement of nutrient acquisition including iron, phosphorus, and nitrogen elements, and production of 1-aminocyclopropane-1-carboxylic acid (ACC) deaminase [20,21]. It was reported that endosymbiont *Piriformospora indica* could promote tomato growth by elevating putrescine, IAA, and GA levels [22]. Moreover, plant-beneficial fungi also indirectly assist plant growth by inhibiting phytopathogen and inducing systemic resistance [23]. For example, *Trichoderma harzianum* greatly reduced the severity of disease caused by the root-knot nematode and therefore contributed to tomato developments [24,25].

However, few studies investigated the beneficial fungi with plant growth-promoting (PGP) traits isolated from mulberry trees to effectively assist host plant growth and enhance drought stress resistance. Therefore, the objectives of this work are as follows: (i) isolate fungi from mulberry trees after suffering drought stress and assess their PGP traits, (ii) detect the effects of plant growth-promoting fungi on mulberry growth and fungal microbiome in rhizosphere soil, and finally (iii) explore the capability of plant growth-promoting fungi on mulberry drought tolerance through pot experiments.

## 2. Results

### 2.1. Isolation and Screening of Plant Growth-Promoting Fungi

A total of 64 isolates were purified from different tissues of well-growing mulberry which suffered periodical drought stress (Table S1), in which 21, 32, and 11 isolates were obtained from stems, roots, and rhizosphere soils, respectively. The dominant phylum was Ascomycota (60 of the 64 isolates, 93.75%), followed by Mucoromycota (4.69%) and Basidiomycota (1.56%), all of which were affiliated with 22 genera. Among them, Nectriaceae (36.67%) and Didymellaceae (15.00%) are the most frequently detected taxonomic families in the 60 Ascomycota isolates. The families Mortierellaceae and Polyporaceae were only detected in Mucoromycota and Basidiomycota, respectively.

A total of sixteen isolates exhibited plant growth-promoting (PGP) traits including indole-3-acetic acid (IAA) and 1-aminocyclopropane-1-carboxylic acid (ACC) deaminase production, and phosphorus solubilization (Table 1). Nine isolates were confirmed to dissolve phosphate, eight isolates possessed ACC deaminase production ability, and four isolates could produce IAA. In order to construct the synthetic fungal suspension, isolates exhibiting PGP traits with complementary function and non-antagonistic interaction were considered for further research. Finally, four isolates were screened out as plant growth-promoting fungi (PGPF) (Table 1). Among them, GR21 and GRs12 accumulated the highest IAA at concentrations of 0.06 mg/mL and 1.22 mg/mL at 4 and 12 days of incubation, respectively (Figure S1A). GS1, GRs12, and GR19 solubilized substantial phosphorous, which exhibited the greatest phosphorous-solubilizing capacity at 10, 18, and 14 days, yielding 0.69, 0.43, and 0.03 mg/mL PO_4_^2−^, respectively (Figure S1B). Moreover, GR21, GRs12, and GS1 also produced ACC deaminase, exhibiting 0.31, 0.86, and 0.45 U/mg activity, respectively (Figure S1C,D).

### 2.2. Identification of the Plant Growth-Promoting Fungi

**Figure 1:**
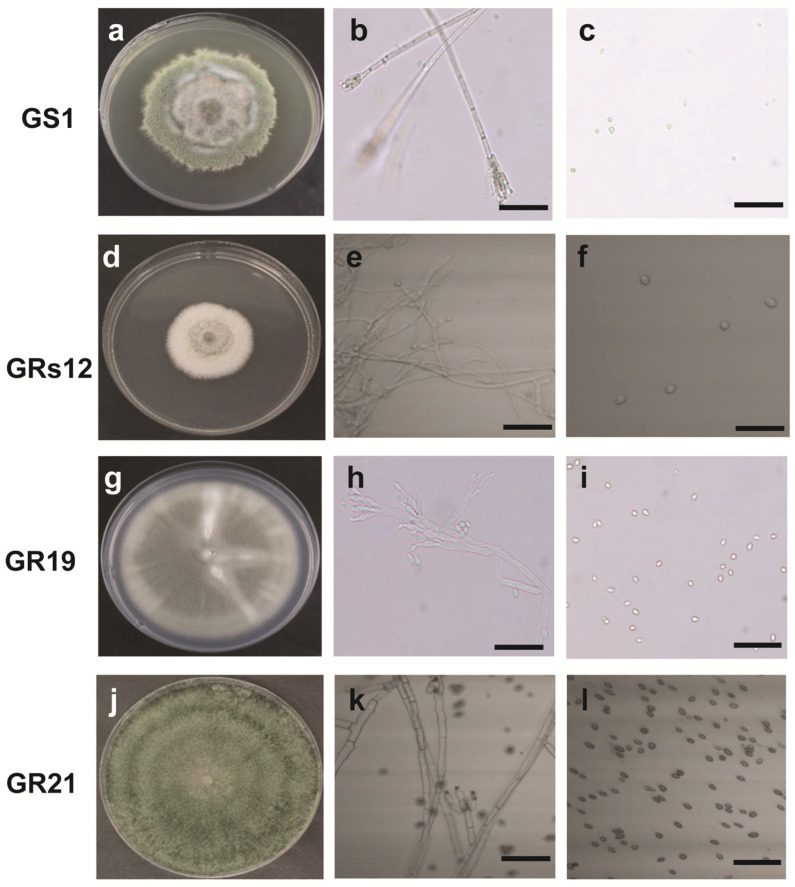
The fungal morphological characteristics of plant growth-promoting fungi in potato dextrose agar at 25 °C after 18 days. (**a**): Colony characteristic of GS1; (**b**): Hyphae morphology of GS1; (**c**): Spore morphology of GS1; (**d**): Colony characteristic of GRs12; (**e**): Hyphae morphology of GRs12; (**f**): Spore morphology of GRs12; (**g**): Colony characteristic of GR19; (**h**): Hyphae morphology of GR19; (**i**): Spore morphology of GR19; (**j**): Colony characteristic of GR21; (**k**): Hyphae morphology of GR21; (**l**): Spore morphology of GR21. The bar represented 25 μm.

The morphological and molecular characteristics of four fungi were determined to identify their taxa. The colony of GS1 grown on potato dextrose agar (PDA) had a villous and rough surface with green color, irregular margin mycelium, and the vegetative mycelia were colorless with the diaphragm (Figure 1a,b). Moreover, a symmetrical small stalk was formed in the shape of a broom at the end of mycelia, wherein round and green spores exist in the branch sporophyll (Figure 1b,c). The colony of GRs12 was flat, white, and cottony with a regular margin (Figure 1d). The mycelium of GRs12 was colorless with branches (Figure 1e), and its spores were round and colorless (Figure 1f). The colony of GR19 had a flat growth surface with an olive green to grey color and regular margin (Figure 1g). The hyphae have septum and form broom bodies with globose conidia (Figure 1h,i). The colony of GR21 was green with a rough growth surface with concentric wheel grain and possessed dense mycelia with green pigment (Figure 1j). The hyphae of GR21 were colorless and septum (Figure 1k), and the spores were round and dark brown (Figure 1l).

**Figure 2:**
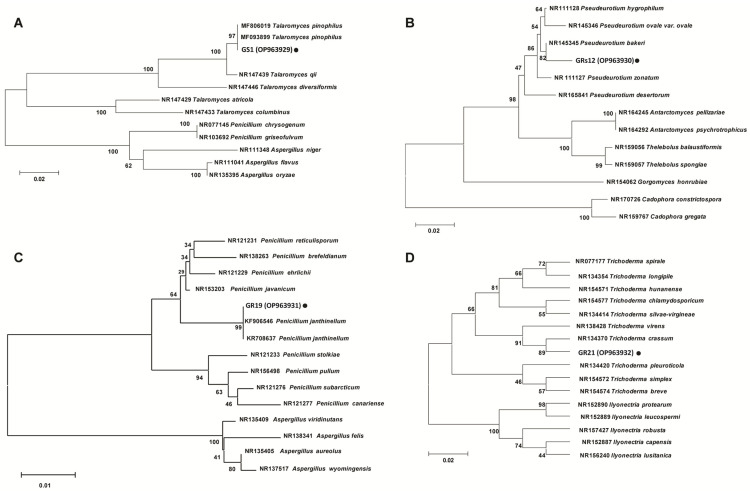
Phylogenetic trees of plant growth-promoting fungi based on internally transcribed spacer sequence. (**A**) GS1 strain. (**B**) GRs12 strain. (**C**) GR19 strain. (**D**) GR21 strain. The trees were constructed by MEGA 6.0 using neighbor-joining analysis of 1000 bootstrap replications.

The phylogenetic trees based on internally transcribed spacer (ITS) sequences showed that the strains of GS1, GRs12, GR19, and GR21 were closely related to *Talaromyces* sp. (Figure 2A), *Pseudeurotium* sp. (Figure 2B), *Penicillium* sp. (Figure 2C), and *Trichoderma* sp. (Figure 2D), respectively. Comprehensively, GS1, GRs12, GR19, and GR21 were, respectively, identified as *Talaromyces* sp. GS1, *Pseudeurotium* sp. GRs12, *Penicillium* sp. GR19, and *Trichoderma* sp. GR21 based on morphological and molecular characteristics.

### 2.3. Plant Growth-Promoting Fungi Facilitated Mulberry Development

**Figure 3:**
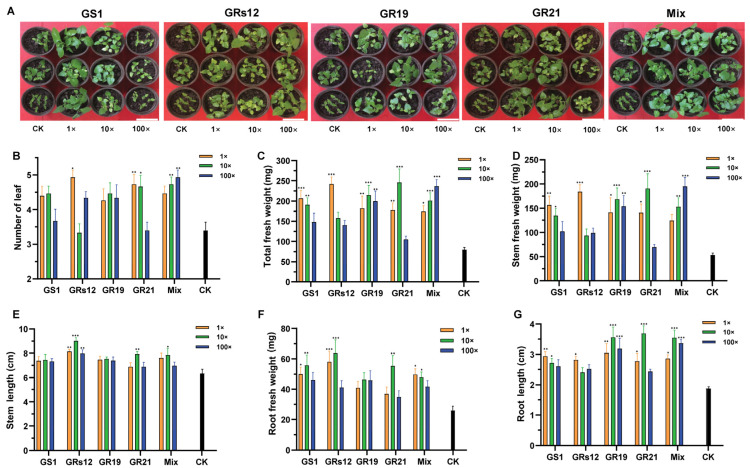
The growth-promoting effects of PGPF on mulberry seedlings. (**A**) The representative photograph of mulberry seedlings after 42 days inoculated with fungal suspensions. The bar represented 10 cm. (**B**) Number of mulberry leaves. (**C**) Total fresh weight of mulberry. (**D**) Fresh weight of mulberry stems. (**E**) Length of mulberry stems. (**F**) Fresh weight of mulberry roots. (**G**) Length of mulberry roots. 1×, Mulberry treated with the initial concentration of fungal suspensions; 10×, Mulberry treated with the 10-fold dilution of fungal suspensions; 100×, Mulberry treated with the 100-fold dilution of fungal suspensions. GS1, GRs12, GR19, GR21, Mix, and CK represented mulberry seedlings treated with *Talaromyces* sp. GS1, *Pseudeurotium* sp. GRs12, *Penicillium* sp. GR19, *Trichoderma* sp. GR21, mixed fungal suspension, and water, respectively. The black bars represented mulberry groups treated with water. Values represented the mean ± standard deviation of replicates (*n* = 15). Statistically significant differences between fungi-treated and control groups were analyzed using Dunnett’s one-way analysis of variance. *** *p* < 0.001, ** *p* < 0.01, * *p* < 0.05.

To investigate the effects of four fungi on mulberry growth, the different concentrations of single and mixed fungal suspensions were inoculated in mulberry seedlings. The co-cultivation results revealed that the growth of mulberry was prompted by fungi (Figure 3A), wherein the leaf numbers and total fresh weights of mulberry were variously increased regardless of single-inoculation or mixed-inoculation (Figure 3B,C). Specifically, the application of *Talaromyces* sp. GS1 strain significantly increased the fresh weight of mulberry stems (*p* < 0.05) (Figure 3D), which was enhanced by 65.83% and 60.24% at the initial concentration and 10-fold diluted suspensions, respectively. Similarly, mulberry seedlings treated with *Talaromyces* sp. GS1 had a higher stem length than the control, increasing by 36.17% and 30.99%, respectively (Figure 3E). In addition, *Pseudeurotium* sp. GRs12 strain significantly assisted mulberry to accumulate more weight and enhance the height of stems only at the initial concentration, which increased by 70.92% and 33.45%, respectively (Figure 3D,E). Mulberry seedlings applied with *Penicillium* sp. GR19 and mixed fungal suspensions at all of the concentrations exhibited more weights and higher lengths of stems than the control (*p* < 0.05) (Figure 3D,E). Moreover, initial concentration and 10-fold diluted suspensions of *Trichoderma* sp. GR21 also significantly promoted mulberry stems growth, wherein the fresh weights were enhanced by 61.95% and 88.99% (Figure 3D), and the lengths were increased by 32.49% and 49.18%, respectively (Figure 3E). Finally, the mixed fungal suspensions at all of the concentrations could promote mulberry stems elongation (Figure 3E) and also contribute to the accumulation of the stem fresh weights (Figure 3D).

Except for the growth promotion of stems, these fungi were beneficial to root growth (Figure 3F,G). All of the single fungi and mixed fungal suspensions enhanced root fresh weights, while the differences between *Penicillium* sp. GR19 and control were not statistically significant. Among them, *Pseudeurotium* sp. GRs12 had the strongest ability to facilitate root developments, wherein fresh weights were enhanced by 55.29% and 59.25% at the initial concentration and 10-fold diluted suspensions, respectively (Figure 3F). Moreover, *Pseudeurotium* sp. GRs12 could also significantly increase the lengths at all of the concentrations (*p* < 0.01), whereas *Talaromyces* sp. GS1 and *Penicillium* sp. GR19 exhibited no obvious effect on root lengths (Figure 3G). However, *Talaromyces* sp. GS1 could prompt the biomass of root at the initial concentration and 10-fold diluted suspensions, enhancing by 48.13% and 53.48%, respectively (Figure 3F). The fresh weights and lengths of root were positively affected by *Trichoderma* sp. GR21 at the 10-fold diluted suspension, which were increased by 53.17% and 20.08%, respectively (*p* < 0.01) (Figure 3F,G). In addition, the mixed fungi also promoted root growth, wherein the fresh weights of root were enhanced by 47.85% and 45.87% at the initial concentration and 10-fold diluted suspensions (Figure 3F). However, the root length of mulberry treated with the mixed fungi has shown no significant difference from that in control at the initial concentration and 100-fold diluted suspensions (Figure 3G). Overall, these results indicated that *Talaromyces* sp. GS1, *Pseudeurotium* sp. GRs12, *Penicillium* sp. GR19, and *Trichoderma* sp. GR21 strains had great potential abilities to promote mulberry development, especially for stems.

### 2.4. Plant Growth-Promoting Fungi Affected Fungal Microbiome of Mulberry Rhizosphere Soil

**Figure 4:**
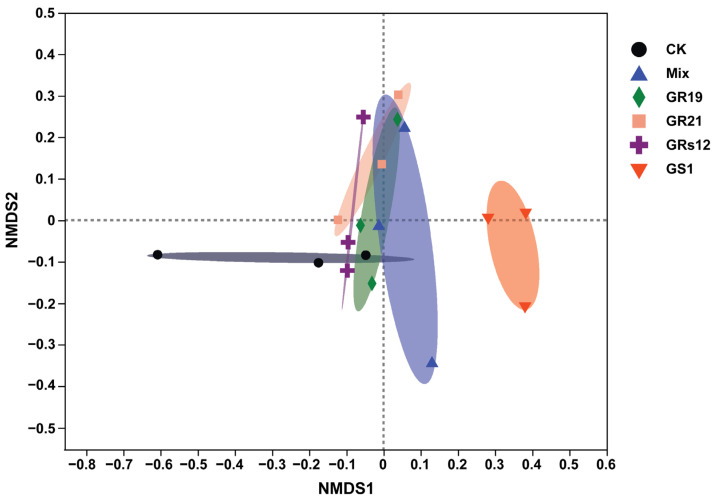
The non-metric multidimensional scaling analysis of fungal communities of mulberry rhizosphere soils using Bray-Curtis distances based on the genus level. GS1, GRs12, GR19, GR21, Mix, and CK represented mulberry seedlings treated with *Talaromyces* sp. GS1 at the initial concentration, *Pseudeurotium* sp. GRs12 at the initial concentration, *Penicillium* sp. GR19 at the 10-fold diluted suspensions, *Trichoderma* sp. GR21 at the 10-fold diluted suspensions, mixed fungi at the 100-fold diluted suspensions, and water, respectively.

In order to examine the influences of PGPF on the fungal microbiome of mulberry rhizosphere soils, the ITS amplicon sequences of fungi in rhizosphere soils of mulberry seedlings with great growth promotion effects in each fungi treatment were conducted (*Talaromyces* sp. GS1 and *Pseudeurotium* sp. GRs12 at the initial concentration; *Penicillium* sp. GR19 and *Trichoderma* sp. GR21 at the 10-fold diluted suspension; mixed fungi at the 100-fold diluted suspension). After quality control and filtering, Illumina MiSeq sequencing analysis produced 1,021,996 reads, representing 355 fungal operational taxonomic units (OTUs) at 97% sequence similarity. The rarefaction curves based on the Sobs index tended to reach a plateau, indicating the depth of sequencing was sufficient (Figure S2). The result of α-diversity analysis indicated the Chao index of fungi was decreased in *Talaromyces* sp. GS1 (49.17) and *Trichoderma* sp. GR21 (74.17), but these groups inoculated with *Pseudeurotium* sp. GRs12 (102.48), *Penicillium* sp. GR19 (111.00), and Mix (117.83) were similar to the control (108.3) (Figure S3A). On the contrary, the Simpson index was increased in *Talaromyces* sp. GS1 (0.68) and *Trichoderma* sp. GR21 (0.59) inoculated groups compared with the control group (0.53), whereas groups of *Pseudeurotium* sp. GRs12 (0.40), *Penicillium* sp. GR19 (0.31), and Mix (0.36) were slightly decreased (Figure S3B). However, the statistical differences in α-diversity between fungi-treated and control groups were not significant (*p* > 0.05). To further compare the relationship of fungal populations among the mulberry rhizosphere soils, the β-diversity shown as non-metric multidimensional scaling (NMDS) and principal coordinate analysis (PCoA) was conducted. The results graphically demonstrated that the application of different fungi was a main factor in accounting for the observed variations in the composition of the fungal community, although the statistical differences of groups were not significant according to Adonis analysis (R^2^ = 0.38; *p* = 0.06). In general, fungal communities mainly formed two separate clusters wherein *Talaromyces* sp. GS1 treatment group was separated from other groups (Figure 4). Fungal communities also clustered in two groups with the distances between inoculated samples being smaller than between controls, except the replicates for *Penicillium* sp. GR19 and Mix (Figure S4A). Moreover, the results obtained by the NMDS analysis were also supported by PCoA (Figure S4B), wherein the distances of inoculated groups were closer than the control groups.

**Figure 5:**
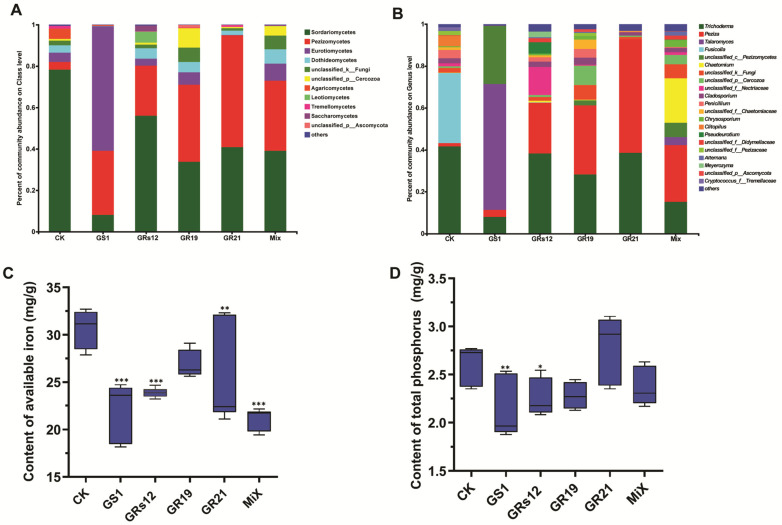
Influence of PGPF on fungal community and physicochemical properties of mulberry rhizosphere soil. (**A**) The fungal community composition at the class level. (**B**) The fungal community composition at the genus level. Taxa with an abundance < 0.01 were included in “others.” Each column represented the mean of three biological replicates. (**C**) Contents of available iron in soils. (**D**) Contents of total phosphorus in soils. GS1, GRs12, GR19, GR21, Mix, and CK represented mulberry seedlings treated with *Talaromyces* sp. GS1 at the initial concentration, *Pseudeurotium* sp. GRs12 at the initial concentration, *Penicillium* sp. GR19 at the 10-fold diluted suspensions, *Trichoderma* sp. GR21 at the 10-fold diluted suspensions, mixed fungi at the 100-fold diluted suspensions, and water, respectively. Values represented the mean ± standard deviation of replicates (*n* = 6). Statistically significant differences between fungi-treated and control groups were analyzed using Dunnett’s one-way analysis of variance. *** *p* < 0.001, ** *p* < 0.01, * *p* < 0.05.

To further understand the influence of four fungi on the shifts of microbial communities, the relative abundance of various fungal taxa was analyzed. Overall, two members of Sordariomycetes and Pezizomycetes were the predominant fungal classes in all fungi-treated samples, whose relative abundance altogether exceeded 70%, except for *Talaromyces* sp. GS1-treated group (31%) (Figure 5A). Notably, Sordariomycetes was the dominant taxon in the control group (78.34%), and its abundance was higher than that in fungi-treated samples (GS1, 8.14%; GRs12, 55.99%; GR19, 33.82%; GR21, 40.81%; Mix, 39.02%). The class of Pezizomycetes was enhanced in rhizosphere soils of mulberry after fungi treatments (GS1, 30.98%; GRs12, 24.23%; GR19, 37.26%; GR21, 54.08%; Mix, 34.01%), but the control group only occupied for 3.72%. Moreover, the rhizosphere soil of *Talaromyces* sp. GS1-treated plants mainly recruited more abundant Eurotiomycetes (60.12%) than other groups (CK, 4.49%; GRs12, 3.34%; GR19, 5.99%; GR21, 0.15%; Mix, 8.1%) (Figure 5A). At the genus level, the most common genera were *Trichoderma* (41.66%) and *Fusicolla* (33.46%) in the control group, whereas *Talaromyces* sp. GS1-treated group mainly accumulated with *Talaromyces* (60.08%) (Figure 5B). Additionally, the genera of *Trichoderma* and *Peziza* were dominant in *Pseudeurotium* sp. GRs12, *Penicillium* sp. GR19, *Trichoderma* sp. GR21, and Mix groups. In particular, the member of *Peziza* in fungi-treated groups (GRs12, 24.19%; GR19, 33.02%; GR21, 54.04%; Mix, 27.08%) showed a higher relative abundance than that in the control (1.61%). It was worth noting that the genus of *Chaetomium* (21.15%) mainly appeared in the Mix group but it was hardly detected in other groups (Figure 5B).

Soil property analysis revealed that both single fungi treatment and mixed fungi treatment could significantly decrease the contents of available iron in soils except for *Penicillium* sp. GR19 (Figure 5C). The available iron in soils was decreased by 27.67%, 22.07%, 17.13%, and 31.04% in *Talaromyces* sp. GS1, *Pseudeurotium* sp. GRs12, *Trichoderma* sp. GR21, and Mix groups (*p* < 0.01). Consistently, soils of *Talaromyces* sp. GS1 and *Pseudeurotium* sp. GRs12 groups possessed lower contents of total phosphorus compared with the control, decreasing by 18.84% and 13.85%, respectively (*p* < 0.05) (Figure 5D). These results suggested fungi might promote mulberry utilization of iron and phosphorus from soils. Furthermore, the Spearman correlation of the top 50 fungi genera with mulberry growth indexes was further investigated to uncover the role of these genera. The heatmap revealed that there was a positive relationship between partial fungi and mulberry development, especially for *Peziza*, *unclassified-p-Cercozoa*, *unclassified-p-Ascomycota*, *Pyrenochaetopsis*, *Cladorrhinum*, *Spizellomyces*, *Arthrinium*, and *Pleurotus* (Figure S5). The results suggested four fungi might attribute to mulberry growth by affecting the fungal microbiome of rhizosphere soil.

### 2.5. Plant Growth-Promoting Fungi Improved Mulberry Drought Tolerance

To determine the synergistic effects of PGPF on the drought tolerance of mulberry seedlings, two types of mixed fungal suspensions (M1: Initial suspensions of *Talaromyces* sp. GS1 and *Pseudeurotium* sp. GRs12, and 10-fold dilution suspensions of *Penicillium* sp. GR19 and *Trichoderma* sp. GR21; M2: 100-fold dilution suspensions of mixed fungi) were employed, and the anti-stress substances were further evaluated at different times. The activities of catalase (CAT) were greatly increased by 234.51% and 139.78% in mulberry treated with M1 and M2 (*p* < 0.05), respectively, than that in the control on the first day of drought stress (Figure S6A). However, no significant differences were observed between fungi-treated plants compared with control plants on the fifth day of drought stress. Moreover, the fungi-treated plants accumulated more concentrations of soluble sugar and chlorophyll compared with control plants. The soluble sugar contents were improved by 25.68% and 28.19% in mulberry applied with M1 and M2 (*p* < 0.05) on the fifth day of drought stress, respectively (Figure S6B). However, only the M1 could significantly increase the content of soluble sugar on the first day of drought stress which was enhanced by 28.64% (*p* < 0.01) (Figure S6B). In addition, the chlorophyll contents (single-photon avalanche diode (SPAD) units) of mulberry were enhanced by 15.06% and 18.01% in M1 and M2 groups (*p* < 0.05), respectively, on the fifth day of drought stress (Figure S6C), but no differences were detected on the first day of drought stress regardless of M1 or M2 treatments.

**Figure 6:**
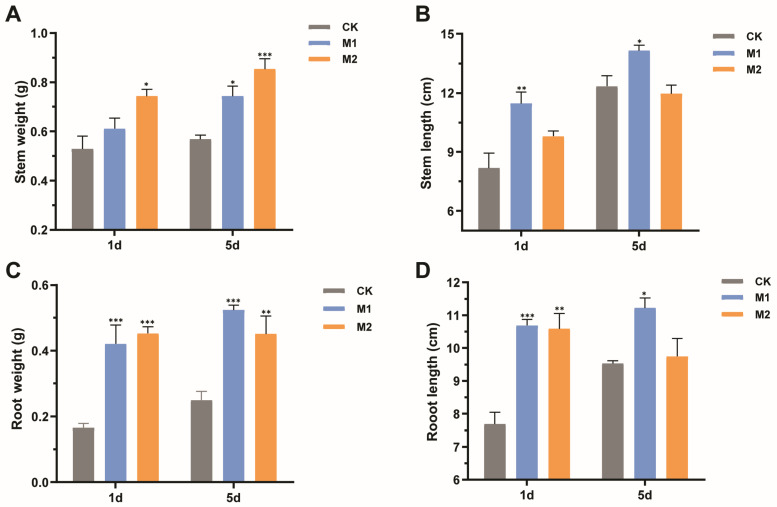
Effects of two types of mixed fungal suspensions on mulberry growth recovering eight weeks after suffering from drought stress. (**A**) Stem fresh weight. (**B**) Stem length. (**C**) Root fresh weight. (**D**) Root length. M1 represented mulberry seedlings treated with mixed fungi suspensions consisting of *Talaromyces* sp. GS1 and *Pseudeurotium* sp. GRs12 at the initial concentration, and *Penicillium* sp. GR19 and *Trichoderma* sp. GR21 at the 10-fold diluted suspensions. M2 represented mulberry seedlings treated with mixed fungi at the 100-fold dilution suspensions. CK represented mulberry seedlings treated with water. Data represent mean ± standard deviation (*n* = 4). Statistically significant differences between fungi-treated and control mulberry seedlings suffering 1 day and 5 days of drought stress were, respectively, analyzed using Dunnett’s one-way analysis of variance. *** *p* < 0.001, ** *p* < 0.01, * *p* < 0.05.

Mulberry seedlings suffering drought stress after 1 day and 5 days were irrigated with water to determine their recovery ability. After an eight-week recovery period, mulberry seedlings treated with M2 had more fresh weights of stems compared with the control (Figure 6A), increasing by 28.94% and 34.12% in mulberry seedlings suffering drought stress after 1 day and 5 days, respectively. Whereas, the application of M2 had no obvious effect on the stem length of mulberry (Figure 6B). Moreover, the stem fresh weight was significantly enhanced from 0.57 g to 0.74 g (*p* < 0.01) in M1-treated mulberry suffering drought stress after 5 days (Figure 6A). Additionally, the M1 group exhibited an obviously positive effect on the stem length of mulberry, increasing by 28.53% and 12.80% in mulberry suffering drought stress after 1 day and 5 days, respectively (*p* < 0.05) (Figure 6B).

In addition, mulberry seedlings treated with mixed fungal suspensions had more root weights and longer lengths than the controls. The root fresh weights were significantly increased by 61.90% and 64.44% in M1- and M2-treated plants suffering drought stress after 1 day (*p* < 0.001) (Figure 6C). An increase of root fresh weights was detected in groups treated with M1 (53.85%) and M2 suspensions (46.67%) suffering drought stress after 5 days (Figure 6C). Similarly, the growth promotion of root length was observed in mulberry seedlings applied with fungi. The M1 suspension facilitated root length, increasing by 28.09% and 27.41% in mulberry suffering drought stress after 1 day and 5 days, respectively. However, M2 suspension only prompted the root length of mulberry suffering drought stress after 1 day (Figure 6D). Collectively, plant-beneficial fungi could accumulate anti-stress substances to improve mulberry resistance to drought and further accelerate mulberry growth recovery.

## 3. Discussion

In the present study, a total of sixty-four fungal isolates were obtained from mulberry. Among them, *Penicillium* sp. GR19, *Trichoderma* sp. GR21, *Talaromyces* sp. GS1, and *Pseudeurotium* sp. GRs12 strains were screened out because of their strong IAA, ACC deaminase production, or phosphate solubilization abilities (Table 1 and Figure S1). Emerging evidences suggest that fungi in the genera of *Trichoderma* spp., *Talaromyces* spp., and *Penicillium* spp. were the most extensively studied taxa and have been shown to benefit plant developments in abiotic stress conditions [26,27,28]. *Trichoderma harzianum* TH1 elevated the growth of rice in drought-stressed soils by triggering various plant metabolic pathways related to anti-oxidative defense, secondary metabolite, and hormonal upregulations [28]. Moreover, *Talaromyces omanensis* enhanced the drought tolerance of tomatoes by improving reproductive, physiological, and anatomical characteristics [29]. The symbiotic association of fungal endophytes *Penicillium brevicompactum* and *Penicillium chrysogenum* with strawberry roots also resulted in a greater shoot and root biomass production, higher fruit number, and an enhanced plant survival rate under water-limiting conditions [30]. Hence, the isolates *Penicillium* sp. GR19, *Trichoderma* sp. GR21, *Talaromyces* sp. GS1, and *Pseudeurotium* sp. GRs12 might play crucial roles in endowing mulberry beneficial traits in drought stress conditions.

The plant growth-promoting effects of four fungi are investigated *in planta* by co-cultivation experiments. The application of *Penicillium* sp. GR19, *Trichoderma* sp. GR21, *Talaromyces* sp. GS1, and *Pseudeurotium* sp. GRs12 strains did significantly enhance the biomass and length of mulberry seedlings, which may be the performance of PGP traits of fungi on mulberry. Naraghi et al. [26] reported, like *Talaromyces* sp. GS1, fungus *Talaromyces flavus* stimulated the growth of cotton and potato which had an increase in root length, crown length, and plant dry weight under greenhouse conditions. *Penicillium oxalicum* UOM PGPF 16 strain isolated from the rhizosphere soil of pearl millet also remarkably enhanced the vegetative growth parameters compared to the control, and improved resistance of pearl millet against downy mildew disease by inducing peroxide and chitinase activities [31]. In addition, Scudeletti et al. [32] revealed that sugarcane plants inoculated with *Trichoderma asperellum* had higher photosynthesis rate, stomatal conductance, and water use efficiency in drought conditions since this strain induced plants to produce higher superoxide dismutase and peroxidase enzyme activities, as well as proline and sugar concentrations. Collectively, these findings suggested *Penicillium* sp. GR19, *Trichoderma* sp. GR21, *Talaromyces* sp. GS1, and *Pseudeurotium* sp. GRs12 could serve as potential agents for bio-fertilizer in agriculture. However, fungal inoculation commonly uses spores rather than mycelium due to their strong vitality. Hence, the method of fungal inoculation using spores should be considered in the future. Moreover, although these fungi exhibited excellent plant growth ability in mulberry, their biosafety should be systemically investigated by genomic analysis and animal assessment.

Inoculation of exogenous microorganisms not only influences the microbiome structures of rhizosphere soil of plants, but recruits many taxa attributed to plant developments [33]. We explored the fungal shifts in the rhizosphere soil of mulberry and found that β-diversity was influenced by *Penicillium* sp. GR19, *Trichoderma* sp. GR21, *Talaromyces* sp. GS1, and *Pseudeurotium* sp. GRs12 (Figure 4), whereas there was little effect on α-diversity (Figure S3). Moreover, the relative abundance of *Fusicolla* was decreased in the rhizosphere soils applied with exogenous fungi. However, the amount of *Peziza* served as ectomycorrhizal fungus was enhanced compared with the control, which preferred to live in disturbed sites [34]. This indicates that there might be a strong disturbance in the rhizosphere due to the addition of PGPF. Interestingly, the application of *Talaromyces* sp. GS1 resulted in a strong enhancement of *Talaromyces* which exceed 60% of relative abundance, indicating *Talaromyces* sp. GS1 possessed a dominantly ecological niche in the rhizosphere soil. Moreover, it is reported that the genus of *Talaromyces* was frequently isolated from soil and served as biological control and bio-fertilizer agent [26,29]. These results showed that *Talaromyces* sp. GS1 could be a great potential candidate for promoting growth and enhancing biotic or abiotic stress tolerance in mulberry. Additionally, it is worth mentioning that the mixed suspensions of four fungi largely recruited the *Chaetomium*. Haruma et al. [34] reported that *Chaetomium* harboring IAA and siderophore production ability could promote plant growth and detoxify aluminum in *Miscanthus sinensis* at the acidic mine site. Hence, the mainly altered fungus *Chaetomium* might thus contribute to mulberry growth. However, the core fungi altered by PGPF need to be isolated and further explored for their promoting efficiency in the future. Furthermore, in the process of fungi-assisted plant growth promotion, some material exchange, energy transformation, and information communication continuously take place among microbes, plant roots, and the soil environments [35]. In the present study, the available iron and total phosphorus contents of soils were influenced by fungi, especially *Talaromyces* sp. GS1 and *Pseudeurotium* sp. GRs12, indicating a feedback effect of these strains on soil. The iron and phosphorus elements decreased by four fungi might be absorbed by mulberry trees and thus benefit their growth. The application of plant growth-promoting fungi not only changed the fungal microbiome but altered the bacterial community in rhizosphere soils. The *Quercus aliena* recruited more abundant bacterial genera *Pedomicrobium*, *Variibacter*, and *Woodsholea* and fungal genera *Aspergillus*, *Phaeoacremonium*, and *Pochonia* in rhizosphere after inoculation with beneficial fungus *Tuber indicum* [36]. Moreover, inoculation with endophytes *Acrocalymma vagum*, *Paraboeremia putaminum*, and *Trichoderma viride* also positively increased Proteobacteria, Actinobacteria, Chloroflexi, and Firmicutes in *Astragalus mongholicus* under drought stress condition [37]. Hence, the bacterial microbiome of mulberry rhizosphere soil treated with four PGPF needs to be further analyzed in the future, which might influence mulberry development as well.

Nowadays, the synthetic community approach attracts more attention because of its functional complementarity [38,39]. For example, Koide [40] suggested that the application of two or more fungi species could bring wider advantages and therefore attribute more benefits to plants. Moreover, Wagg et al. [41] found that plants inoculated with fungi with complementary traits showed more productivity than that in single fungal inoculation. Hence, to perform synergistic effects of four fungi on the growth of mulberry seedlings under drought conditions, we conducted two types of mixed fungal suspensions in co-cultivation experiments. As expected, the mulberry seedlings inoculated with mixed suspensions accumulated more anti-stress substances than the control group such as CAT and soluble sugar, which play important roles in ameliorating damages of abiotic stress [42,43,44]. Similarly, the combined inoculation with *Funneliformis geosporus* and *Funneliformis mosseae* positively promoted growth and enhanced the drought tolerance of strawberries [45]. Moreover, our results also demonstrated the mixed fungi helped mulberry recovery after experiencing drought stress. Therefore, the four fungi possessed an excellent ability to stimulate mulberry growth and enhance drought tolerance. Whereas, it will be necessary to understand how fungi influence mulberry growth under drought-stress conditions using transcriptome and metabolomics, because synthetic fungi might redundantly perform functions in the interactions with host plants. Collectively, the current study offers a potential approach to increase the drought tolerance of mulberry and thereby boost mulberry fruit yields by utilizing beneficial fungi in adverse environments.

## 4. Materials and Methods

### 4.1. Sampling Collection

The rhizosphere soil, root, and stem were collected from well-growing mulberry trees (“Guisangyou 62”, a variety of white mulberry) in May 2018, which suffered about a three-month drought stress each year. These mulberry trees were planted in the hydro-fluctuation belt of the Three Gorges Reservoir area in Longjiao Town, Yunyang County, Chongqing City, China (30°49′25″ N, 108°52′12″ E) in 2012. The height of mulberry tree was about 4 m. Rhizosphere soil adhering to mulberry root of approximately 5 mm was obtained. Roots of 20 cm in length were harvested at about 30 cm below the soil surface, and stems of 25 cm in length were collected at about 1 m height above the ground. Three biological replicates were performed for each sample. All samples were stored at 4 °C until further use.

### 4.2. Isolation and Classification of Mulberry-Associated Fungi

The surface sterilization of stems and roots was performed as described by Xu et al. [46]. Briefly, samples were completely immersed in 75% ethanol and immediately ignited on the alcohol lamp. Then, surface sterilized samples were cut into pieces 3.5∼5.0 cm in length [47], and these samples were peeled to obtain smaller fragments and placed on Czapek dox agar (CZA), Gauze’s agar #1 (GA), oatmeal agar (OA), water agar (WA), and potato dextrose agar (PDA) containing 35 μg/mL streptomycin and cultured at 25 °C. Fungi from rhizosphere soil were isolated via serial dilution. In brief, 1 g of rhizosphere soil was suspended with 30 mL of sterile distilled water and placed in a shaker at 220 rpm for 30 min to make the soil suspension. The soil suspension was serially diluted 10-fold until it reached 1 × 10^−6^. Subsequently, 200 µL suspensions of 10^−4^ to 10^−6^ dilutions were transferred into CZA, GA, OA, WA, and PDA. The different morphological fungi were chosen and purified.

The fungal isolates were identified based on the analysis of internal transcribed spacer (ITS) gene sequencing. The total DNA of the isolate was extracted using the Prep Man Ultra Sample Preparation Reagent kit (Applied Biosystems, Palo Alto, CA, USA) according to the manufacturer’s instructions. The DNA was amplified by PCR using the primers ITS1 (5′-TCCGTAGGTGAACCTGCGG-3′) and ITS4 (5′-TCCTCCGCTTATTGAGATATGC-3′) [48]. The PCR products were sent to Sangon Biotech Co., Ltd. (Shanghai, China) for sequencing. The obtained gene sequences were compared and analyzed in NCBI to determine the taxa of isolated fungi. The isolates were classified at the genus level according to the BLAST results showing > 97% identity [46].

### 4.3. Screening and Identification of Plant Growth-Promoting Fungi

Plant growth-promoting potential traits of isolates were evaluated by testing phosphate solubilization, and indole-3-acetic acid (IAA) and 1-aminocyclopropane-1-carboxylic acid (ACC) deaminase production abilities. Specifically, isolates were inoculated in Pikovskaya’s agar (PVK) containing tricalcium phosphate and cultured at 25 °C for 7 days. The formation of a hydrolytic circle showed that the fungi could dissolve phosphorus [49]. The quantitative phosphate solubilization activity of fungi was determined as previously described by Gaind [50]. The ability to produce IAA was assayed by inoculating the isolates into potato dextrose liquid (PDB) medium containing 0.4 g/L tryptophan at 25 °C for 7 days and centrifuging at 12,000 rpm. Then, 1 mL of Salkowski reagent was added to 500 μL of supernatant, and the solution was kept in the dark for 30 min. The formation of red color in the reaction solution suggested isolates could produce IAA, and their contents were assayed as described by Afzal et al. [51]. In addition, ACC deaminase activity was assayed by Dworkin and Foster (DF) and ADF (DF medium added to 3 mM of ACC) media. Briefly, isolates were inoculated in DF and then transferred into ADF at 25 °C at 180 rpm. After 10 days, the isolates grown normally in ADF showed that they could utilize ACC, and the ACC deaminase activities were detected as previously described by Penrose et al. [52].

Isolates (GS1, GRs12, GR19, and GR21) exhibiting extraordinary plant growth-promoting potentials with complementary function and non-antagonistic interaction in vitro were considered as plant growth-promoting fungi (PGPF) for further research. Fungal isolates were inoculated into PDA and incubated at 22 °C, and then the mycelium morphology was recorded, and the spore shape was further observed under the light microscope. Molecular identification of the fungal isolate was conducted based on the sequence of ITS genes. The obtained sequences were compared with the Genbank database of NCBI, and the sequences with high homology were selected to build phylogenetic trees in MEGA 6.0 software using the neighbor-joining method with 1000 bootstrap replications.

### 4.4. Growth Promotion Assay of Plant Growth-Promoting Fungi in Mulberry

The PGPF were inoculated in PDB and cultured at 25 °C with 180 rpm for 10 days. Afterward, the fungal suspensions were centrifuged at 12,000 rpm for 30 min to obtain precipitation. Approximately 6.8 g of mycelium was homogenized in an aseptic tissue homogenizer using 100 mL of sterile water to obtain an initial suspension. Additionally, the fungal suspensions of each strain at the initial suspension were mixed (*v*/*v*/*v*/*v*/, 1:1:1:1) to obtain mixed fungi (Mix). Afterward, the prepared fungal suspensions including single and mix fungi were serially diluted with sterile water to 10-fold and 100-fold for further process.

The disinfected mulberry seeds (“Guisangyou 12”) were placed in a sterile petri-dish containing moist filter paper and cultivated in a growth chamber at 25 °C with 12 h light (intensity 8000 l×), 12 h dark, and 85% humidity. Each petri-dish contained 20 seeds and was conducted with thirty replicates. At the two-leaf stage, six seedlings with the same growth were selected and planted in one pot containing mixtures of humus and field soil (*v*/*v*, 2:1). Fungi-treated and control groups were conducted with 15 biological replicates.

After one month of cultivation, mulberry seedlings were irrigated with 20 mL of prepared single or mixed fungal suspensions with different concentrations (i.e., the initial concentration, 6.8 × 10^−2^ g/mL; 10-fold dilution, 6.8 × 10^−3^ g/mL; 100-fold dilution, 6.8 × 10^−4^ g/mL). The second treatment was carried out 7 days later. Mulberry seedlings treated with an equal volume of water were served as control. After forty days, the stem/root fresh weights and root/stem lengths of 15 randomly selected mulberry seedlings were detected.

### 4.5. Effects of Plant Growth-Promoting Fungi on Fungal Community Profiles in the Rhizosphere Soil

To explore the potential mechanism of fungi to promote mulberry growth attributed to the variation of the fungal community, the rhizosphere soils of mulberry seedlings with the great growth promotion effects in each fungi treatment were obtained (GS1 and GRs12 at the initial concentration; GR19 and GR21 at the 10-fold diluted suspensions; mixed fungi at the 100-fold diluted suspensions). About 5 g of rhizosphere soils adhering to the root of approximately 5 mm were slightly collected using a sterile scalpel for further fungal microbiome analysis and three replicates were conducted.

Total fungal DNA was extracted from rhizosphere soil sample using the FastDNA^®^ Spin Kit for Soil (MP Bio, Santa Ana, CA, USA) using the manufacturer’s instructions [53]. The ITS sequence of fungi was amplified using primers ITS1F (5′-CTTGGTCATTTAGAGGAAGTAA-3′) and ITS2R (5′-GCTGCGTTCTTCATCGATGC-3′) [46]. Purified amplicons were pooled in equimolar aliquots and sequenced on the Illumina MiSeq platform (Illumina, San Diego, USA) by Majorbio Bio-Pharm Technology Co., Ltd. (Shanghai, China). The paired-end reads were spliced into a sequence and filtered to obtain high-quality reads by quality control. Non-repetitive sequences from optimized sequences were extracted using Usearch software (version 7.0), and single sequences without repetitions were removed. Only sequences with 97% similarity were clustered to the same OTU (operational taxonomic unit) without Chimera using Uparse (version 7.0.1090). Ribosomal Database Project (RDP) classifier Bayesian algorithm (version 2.11) was utilized for the taxonomic analysis of OTU representative sequences according to the Unite database (Release 8.0). Each sample was rarefied to 3000 reads. Mothur software (version 1.30.2) was used to calculate α-diversity including Chao and Simpson indices. Based on the sobs index at the OTU level, the rarefaction curve was performed on each sample to assess the adequacy of the sampling. Non-metric multidimensional scaling (NMDS) and principal coordinate analysis (PCoA) were used to analyze the difference of β-diversity based on the Bray-Curtis distance according to Adonis analysis. The fungal community composition was conducted using the online platform Majorbio Cloud Platform (Available online: www.majorbio.com (accessed on 8 July 2020)). The Spearman correlation coefficient of the top 50 abundant fungal genera and mulberry growth parameters was calculated and displayed using a heatmap.

Furthermore, approximately 50 g of bulk soil samples were also collected from each pot for detection of the available iron and total phosphorus contents, and six replicates were conducted. Soils were dried at 105 °C and filtered with a 2 mm sieve. The total phosphorus content was analyzed using the Mo-Sb antispectrophotometric method as described by the agriculture protocols [54]. Briefly, 0.5 M NaHCO_3_ was added to soils and shaken at 200 rpm at 25 °C. The solutions were immediately filtered and added to 2,4-dinitrophenol and sulfuric acid. Then, ascorbic acid and molybdenum-antimony were added, and the solutions were maintained at 30 °C for 30 min. The optical density of the suspension was measured at 880 nm to assay the total phosphorus content. In addition, DTPA-CaCl_2_-TEA was added to the soils and shaken at 200 rpm at 25 °C for 2 h. Then, the solutions were immediately filtered and their optical densities were measured at 248 nm by atomic absorption spectrophotometry to assay available iron [55].

### 4.6. Effects of Plant Growth-Promoting Fungi on Drought Tolerance of Mulberry Seedling

To detect whether the isolates have synergistic effects on the drought tolerance of mulberry seedlings, two types of mixed fungal suspension were employed in the drought stress experiment. According to the growth promotion effects of four fungi on mulberry seedlings, the concentration of each fungus with the great growth promotion effect was selected (GS1 and GRs12: 6.8 × 10^−2^ g/mL, GR19 and GR21: 6.8 × 10^−3^ g/mL), and each fungal suspension was mixed in equal volume to obtain fungal suspensions M1. Moreover, fungal initial suspensions (6.8 × 10^−2^ g/mL) were mixed in equal volumes and diluted 100-fold to obtain suspensions M2.

The methods of seed germination and seedling inoculation were described above. Fungi-treated and control groups were conducted with 15 biological replicates. After a five-week inoculation, sterile water was irrigated into pots containing mulberry seedlings until the water overflowed. Then, irrigation was stopped and the mulberry seedlings were placed at 25 °C. After 8 days, the mulberry seedlings began to wilt and the time point was recorded as the first day of drought stress. The chlorophyll contents of leaves at the same position as mulberry seedlings were detected using a chlorophyll meter LYS-4N (Lvbo Instrument Co., Ltd., Hangzhou, China) on the first day and the fifth day of drought stress [56]. Then, mulberry leaves were randomly collected and stored at −80 °C for the determination of anti-stress substances [57,58]. The soluble sugar content and catalase activity were analyzed by the biochemical kits according to the manufacturer’s instructions (Comin Biotech Co., Ltd., Suzhou, China) [59,60,61]. The total soluble sugar content was analyzed using the anthrone colorimetry method. Briefly, about 0.1 g of samples were homogenized in 1 mL of distilled water. The suspension was then centrifuged at 10,000 rpm for 10 min. Afterward, the sulfuric acid and anthrone colorimetry were subsequently added and incubated at 95 °C for 10 min. Then, the optical density of solutions was immediately measured at 620 nm to assay soluble sugar contents. In addition, the activity of catalase was analyzed using the ammonium molybdate colorimetric method. About 0.1 g of samples were homogenized in 1 mL of phosphate buffer saline and centrifuged at 10,000 rpm for 10 min. The hydrogen peroxide and ammonium molybdate solution were subsequently added and their optical density was immediately measured at 405 nm to assay catalase activity.

After completion of sampling on the first day and fifth day of drought stress, the remaining mulberry seedlings were immediately cultivated under normal growth conditions with watering every 3 days. After 8 weeks of recovery, the lengths and fresh weights of stems and roots of mulberry seedlings were measured.

### 4.7. Statistical Analysis

All data in the present study were performed using SPSS (version 17.0) and GraphPad Prism (version 8.0.2.263). Differences of mulberry seedlings in growth promotion and drought stress assays, α-diversity of the fungal microbiome of the rhizosphere, and soil biochemical properties between fungi-treatment and control groups were analyzed using one-way analysis of variance, and *, **, and *** indicated significant difference at *p* < 0.05, *p* < 0.01, and *p* < 0.001, respectively. The data were displayed as mean ± standard deviation.

## 5. Conclusions

The yields of mulberry fruits and leaves are usually strongly hindered by drought stress. In the present study, inoculation of four PGPF significantly stimulates mulberry growth in a greenhouse and also increases its drought stress tolerance, which might further boost mulberry fruit production. The beneficial effects of four PGPFs on mulberry growth provide a promising strategy to overcome drought pressure. As an eco-friendly practice, utilization of PGPF in crops is a potential prospect for productivity enhancement in agriculture.

## Figures and Tables

**Table 1:** Characterization of mulberry-associated fungi for potential plant growth-promoting traits.

Isolates	Origin	Taxa	Dissolving Phosphorus	IAA Production	ACC Deaminase
**GS1**	Stem	***Talaromyces* sp.**	+	-	+
GRs7	Rhizosphere soil	*Acrocalymma* sp.	-	+	-
GRs11	Rhizosphere soil	*Mortierella* sp.	-	+	-
**GRs12**	Rhizosphere soil	***Pseudeurotium* sp.**	+	+	+
GR3	Root	*Boeremia* sp.	-	-	+
GR4	Root	*Aspergillus* sp.	-	-	+
GR6	Root	*Aspergillus* sp.	+	-	-
GR9	Root	*Fusarium* sp.	+	-	-
GR12	Root	*Fusarium* sp.	-	-	+
GR14	Root	*Pseudeurotium* sp.	+	-	-
**GR19**	Root	***Penicillium* sp.**	+	-	-
**GR21**	Root	***Trichoderma* sp.**	-	+	+
GR22	Root	*Aspergillus* sp.	+	-	-
GR27	Root	*Fusarium* sp.	-	-	+
GR30	Root	*Pseudeurotium* sp.	+	-	+
GR32	Root	*Pseudeurotium* sp.	+	-	-

Note: “+” means a positive result; “-” means a negative result. The bold isolates were selected as potential plant growth-promoting fungi (PGPF) due to their great growth-promoting traits.

## Data Availability

The ITS gene sequences of four plant growth-promoting fungi were deposited in the GenBank under accession numbers OP963929-OP963932. The raw Illumina sequence data were submitted to the NCBI Short Read Archive (SRA) database under the accession number SRP411931 and the BioProject accession number was PRJNA909945. This data can be found at https://www.ncbi.nlm.nih.gov/sra/PRJNA909945 (accessed on 8 April 2022).

## Supplementary Materials

The following supporting information can be downloaded at: https://www.mdpi.com/article/10.3390/ijms24044090/s1.
