# Supplementary material for: Study on the Potential for Stimulating Mulberry Growth and Drought Tolerance of Plant Growth-Promoting Fungi
Source: Int J Mol Sci. 2023 Feb 17;24(4):4090. doi: 10.3390/ijms24044090 (PMC9966926; doi:10.3390/ijms24044090)
Supplement: Supplementary file 1 [file ijms-24-04090-s001.zip › ijms-2121647-supplementary.pdf]

Supporting information

Table S1 Taxonomic analysis of fungal isolates from mulberry trees.

Strain ID	Source	Phylum	Class	Family	Genus
GS1	stem	Ascomycota	Eurotiomycetes	Trichocomaceae	*Talaromyces*
GS2	stem	Ascomycota	Sordariomycetes	Nectriaceae	*Fusarium*
GS3	stem	Ascomycota	Sordariomycetes	Nectriaceae	*Fusarium*
GS4	stem	Ascomycota	Sordariomycetes	Nectriaceae	*Fusarium*
GS5	stem	Ascomycota	Sordariomycetes	Nectriaceae	*Fusarium*
GS6	stem	Ascomycota	Sordariomycetes	Nectriaceae	*Fusarium*
GS7	stem	Ascomycota	Dothideomycetes	Massarineae	*Lentithecium*
GS8	stem	Ascomycota	Sordariomycetes	Nectriaceae	*Fusarium*
GS9	stem	Ascomycota	Sordariomycetes	Xylariaceae	*Xylaria*
GS10	stem	Ascomycota	Sordariomycetes	Nectriaceae	*Fusarium*
GS11	stem	Ascomycota	Dothideomycetes	Massarineae	*Lentithecium*
GS13	stem	Ascomycota	Sordariomycetes	Nectriaceae	*Fusarium*
GS14	stem	Ascomycota	Dothideomycetes	Didymellaceae	*Phoma*
GS15	stem	Ascomycota	Dothideomycetes	Massarineae	*Aquastroma*
GS16	stem	Ascomycota	Dothideomycetes	Massarineae	*Lentithecium*
GS19	stem	Ascomycota	Sordariomycetes	Nectriaceae	-
GS20	stem	Ascomycota	Sordariomycetes	Clavicipitaceae	*Aschersonia*
GS21	stem	Ascomycota	Sordariomycetes	Nectriaceae	*Fusarium*
GS22	stem	Ascomycota	Sordariomycetes	Diaporthaceae	*Diaporthe*
GS23	stem	Ascomycota	Sordariomycetes	Nectriaceae	*Fusarium*
GS24	stem	Basidiomycota	Agaricomycetes	Polyporaceae	*Trametes*
GR1	root	Ascomycota	Sordariomycetes	Nectriaceae	*Fusarium*
GR2	root	Ascomycota	Sordariomycetes	Nectriaceae	*Fusarium*
GR3	root	Ascomycota	Dothideomycetes	Didymellaceae	*Boeremia*
GR4	root	Ascomycota	Eurotiomycetes	Aspergillaceae	*Aspergillus*
GR5	root	Ascomycota	Sordariomycetes	Ophiocordycipitaceae	*Purpureocillium*
GR6	root	Ascomycota	Eurotiomycetes	Aspergillaceae	*Aspergillus*
GR7	root	Ascomycota	Eurotiomycetes	Aspergillaceae	*Penicillium*
GR8	root	Ascomycota	Dothideomycetes	Didymellaceae	*Juxtiphoma*
GR9	root	Ascomycota	Sordariomycetes	Nectriaceae	*Fusarium*
GR10	root	Ascomycota	Dothideomycetes	Didymellaceae	*Juxtiphoma*
GR11	root	Ascomycota	Dothideomycetes	Didymellaceae	*Juxtiphoma*
GR12	root	Ascomycota	Sordariomycetes	Nectriaceae	*Fusarium*
GR13	root	Ascomycota	Dothideomycetes	Didymellaceae	*Boeremia*
GR14	root	Ascomycota	Leotiomycetes	Pseudeurotiaceae	*Pseudeurotium*
GR15	root	Ascomycota	Sordariomycetes	Nectriaceae	*Ilyonectria*
GR16	root	Ascomycota	Sordariomycetes	Nectriaceae	*Fusarium*
GR17	root	Ascomycota	Dothideomycetes	Didymellaceae	*Juxtiphoma*
GR18	root	Ascomycota	Dothideomycetes	Didymellaceae	*Juxtiphoma*
GR19	root	Ascomycota	Eurotiomycetes	Aspergillaceae	*Penicillium*

GR20	root	Ascomycota	Leotiomycetes	Pseudeurotiaceae	*Pseudeurotium*
GR21	root	Ascomycota	Sordariomycetes	Hypocreaceae	*Trichoderma*
GR22	root	Ascomycota	Eurotiomycetes	Aspergillaceae	*Aspergillus*
GR23	root	Ascomycota	Eurotiomycetes	Aspergillaceae	*Penicillium*
GR24	root	Ascomycota	Dothideomycetes	Cladosporiaceae	*Cladosporium*
GR25	root	Ascomycota	Sordariomycetes	Hypocreaceae	*Trichoderma*
GR26	root	Ascomycota	Sordariomycetes	Nectriaceae	-
GR27	root	Ascomycota	Sordariomycetes	Nectriaceae	*Fusarium*
GR29	root	Ascomycota	Dothideomycetes	Cladosporiaceae	*Cladosporium*
GR30	root	Ascomycota	Leotiomycetes	Pseudeurotiaceae	*Pseudeurotium*
GR32	root	Ascomycota	Leotiomycetes	Pseudeurotiaceae	*Pseudeurotium*
GR33	root	Ascomycota	Sordariomycetes	Nectriaceae	*Fusarium*
GR35	root	Ascomycota	Saccharomycetes	Dipodascaceae	*Galactomyces*
GRs1	rhizosphere soil	Ascomycota	Eurotiomycetes	Aspergillaceae	*Penicillium*
GRs2	rhizosphere soil	Ascomycota	Sordariomycetes	Nectriaceae	*Fusarium*
GRs3	rhizosphere soil	Ascomycota	Dothideomycetes	Didymellaceae	*Juxtiphoma*
GRs4	rhizosphere soil	Mucoromycota	Zygomycetes	Mortierellaceae	*Mortierella*
GRs5	rhizosphere soil	Ascomycota	Sordariomycetes	Bionectriaceae	*Clonostachys*
GRs7	rhizosphere soil	Ascomycota	Dothideomycetes	Morosphaeriaceae	*Acrocalymma*
GRs8	rhizosphere soil	Ascomycota	Sordariomycetes	Nectriaceae	*Fusarium*
GRs9	rhizosphere soil	Ascomycota	Eurotiomycetes	Aspergillaceae	*Aspergillus*
GRs10	rhizosphere soil	Mucoromycota	Zygomycetes	Mortierellaceae	*Mortierella*
GRs11	rhizosphere soil	Mucoromycota	Zygomycetes	Mortierellaceae	*Mortierella*
GRs12	rhizosphere soil	Ascomycota	Leotiomycetes	Pseudeurotiaceae	*Pseudeurotium*

Note: "-" represents unclassified fungi at the genus level.

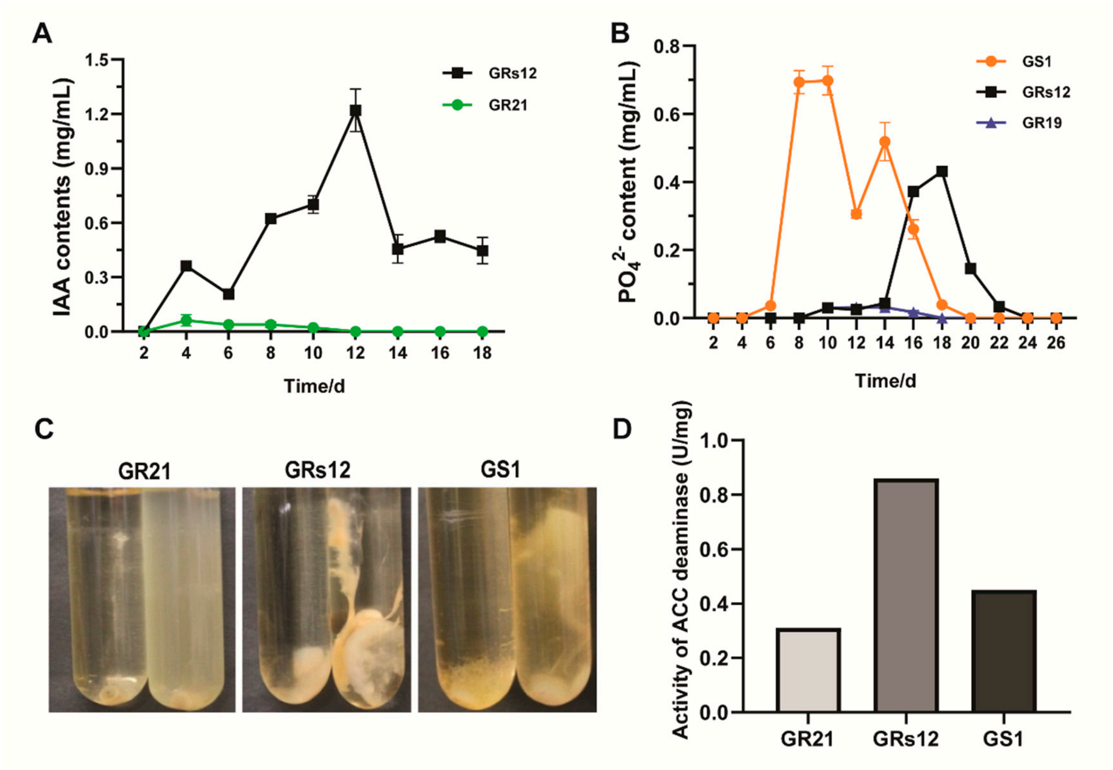

Figure S1. The plant growth-promoting traits of potential plant growth-promoting fungi. (**A**) The IAA production by GRs12 and GR21 strains over time. (**B**) Phosphate from $Ca_3(PO_4)_2$ released by GS1, GRs12, and GR19 strains over time. Data represent mean ± standard deviation (*n* = 3). (**C**) The ability to utilize ACC of GR21, GRs12, and GS1 strains. Left, the control medium without ACC. Right, ADF medium adding with 3 mM ACC. (**D**) The activity of ACC deaminase of GR21, GRs12, and GS1 strains after 10-day inoculation.

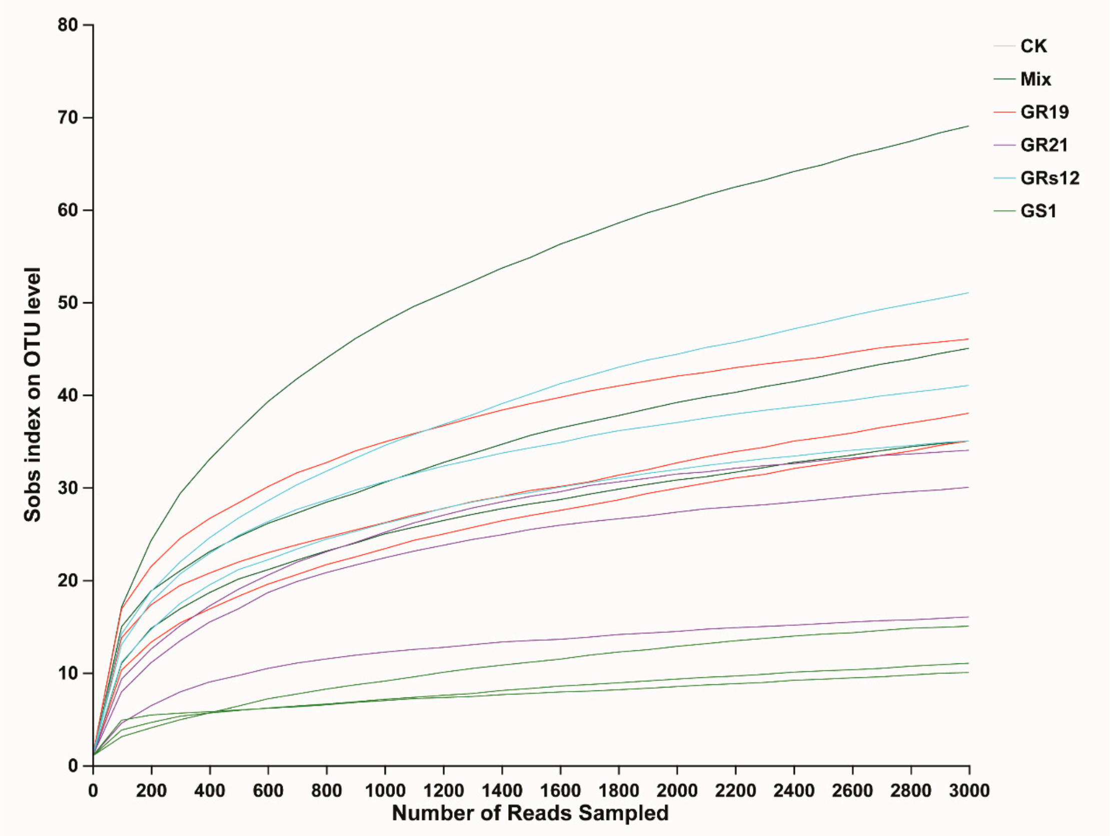

Figure S2. Rarefaction curves depicting the number of OTUs with 97% similarity identified from rhizosphere soil of fungi-treated and control groups. GS1, GRs12, GR19, GR21, Mix, and CK represented mulberry seedlings treated with *Talaromyces* sp. GS1 at the initial concentration, *Pseudeurotium* sp. GRs12 at the initial concentration, *Penicillium* sp. GR19 at the 10-fold diluted suspensions, *Trichoderma* sp. GR21 at the 10-fold diluted suspensions, mixed fungi at 100-fold diluted suspensions, and water, respectively.

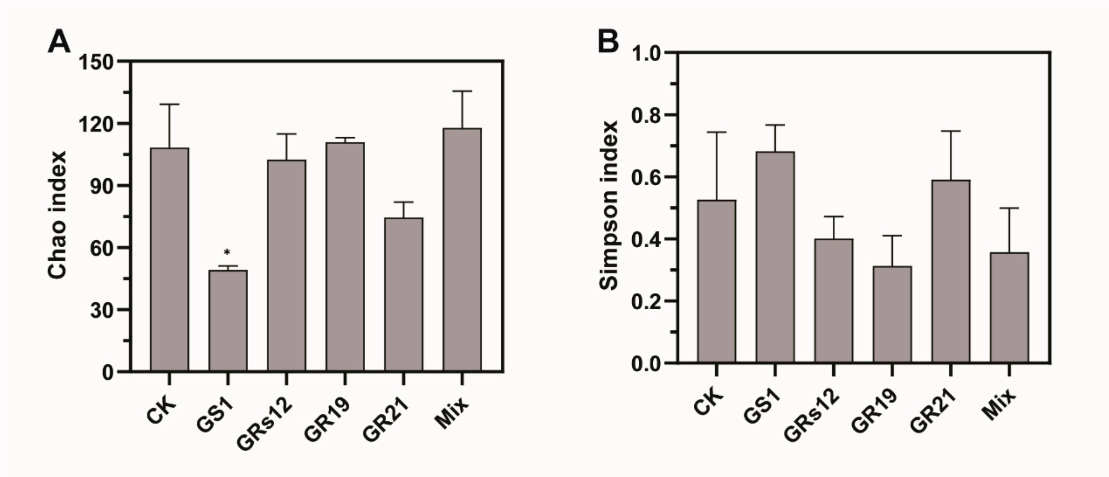

Figure S3. The α-diversity of rhizosphere soil of control and inoculated mulberry. (**A**) The Chao index. (**B**) The Simpson index. Data represented mean ± standard deviation (n = 3). Statistically significant differences between fungi-treated and control groups were analyzed using Dunnett's one-way analysis of variance. * $P < 0.05$. GS1, GRs12, GR19, GR21, Mix, and CK represented mulberry seedlings treated with *Talaromyces* sp. GS1 at the initial concentration, *Pseudeurotium* sp. GRs12 at the initial concentration, *Penicillium* sp. GR19 at the 10-fold diluted suspensions, *Trichoderma* sp. GR21 at the 10-fold diluted suspensions, mixed fungi at the 100-fold diluted suspensions, and water, respectively.

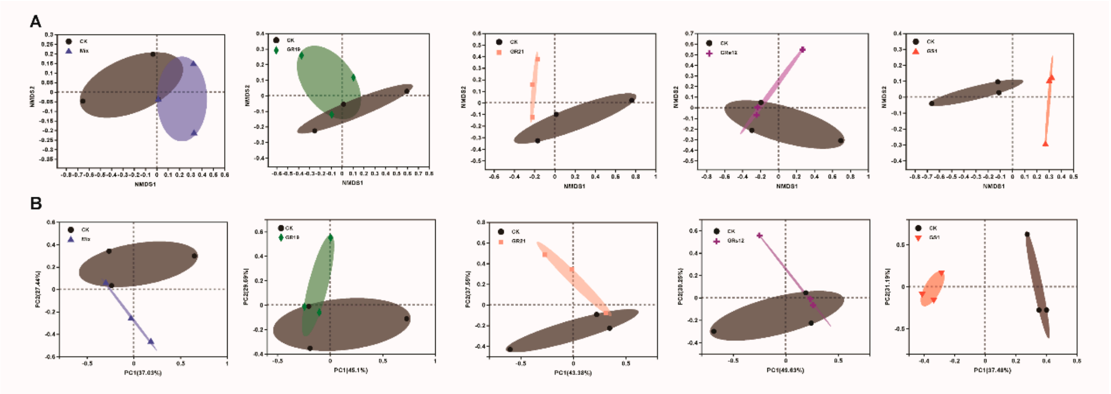

Figure S4. The β-analysis of fungal communities of rhizosphere soil based on Bray-Curtis distances. (**A**) NMDS analysis between plant growth-promoting fungi treated group with control group. (**B**) PCoA analysis between plant growth-promoting fungi treated group with control group. GS1, GRs12, GR19, GR21, Mix, and CK represented mulberry seedlings treated with *Talaromyces* sp. GS1 at the initial concentration, *Pseudeurotium* sp. GRs12 at the initial concentration, *Penicillium* sp. GR19 at the 10-fold diluted suspensions, *Trichoderma* sp. GR21 at the 10-fold diluted suspensions, mixed fungi at the 100-fold diluted suspensions, and water, respectively.

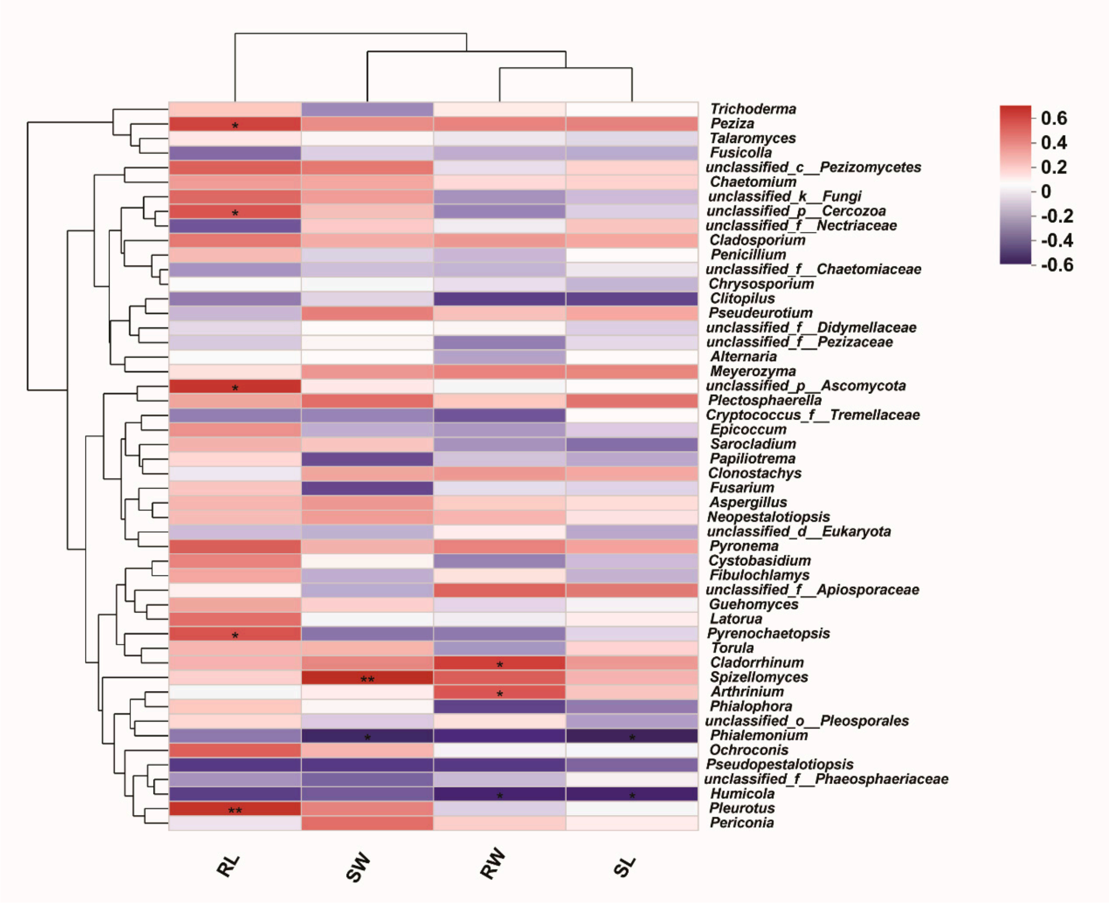

Figure S5. Heatmap of correlation between mulberry growth parameters and top 50 fungal genera using the Spearman correlation coefficient. RL and SL represented the length of mulberry root and shoot, respectively. RW and SW represented the fresh weight of mulberry root and shoot, respectively. ** $P < 0.01$, * $P < 0.05$.

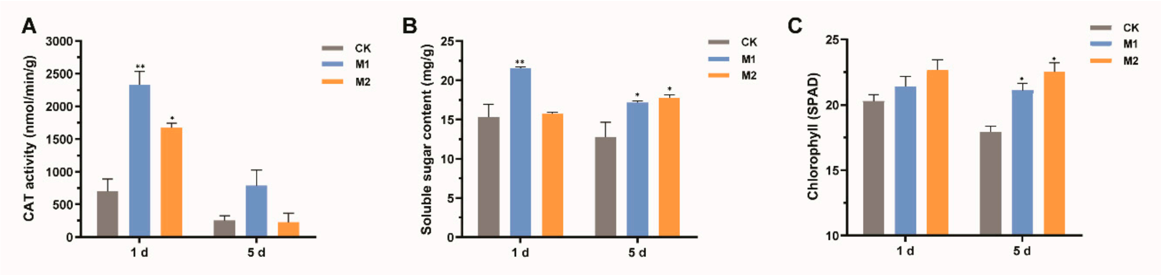

Figure S6. Effects of two types of mixed fungal suspensions on anti-stress substrates of mulberry. (**A**) CAT enzyme activity. (**B**) Soluble sugar content. (**C**) Chlorophyll content. M1 represented mulberry seedlings treated with mixed fungi suspensions consisting of *Talaromyces* sp. GS1 and *Pseudeurotium* sp. GRs12 at the initial concentration, and *Penicillium* sp. GR19 and *Trichoderma* sp. GR21 at the 10-fold diluted suspensions. M2 represented mulberry seedlings treated with mixed fungi at 100-fold dilution suspensions. CK represented mulberry seedlings treated with water. Data represent mean ± standard deviation (n = 3). Statistically significant differences between fungi-treated and control mulberry seedlings suffering 1 day and 5 days of drought stress were respectively analyzed using Dunnett's one-way analysis of variance. ** $P < 0.01$, * $P < 0.05$.
